# It's Not What You Expected! The Surprising Nature of Cleft Alternatives in French and English

**DOI:** 10.3389/fpsyg.2019.01400

**Published:** 2019-06-18

**Authors:** Emilie Destruel, David I. Beaver, Elizabeth Coppock

**Affiliations:** ^1^Department of French and Italian, University of Iowa, Iowa City, IA, United States; ^2^Department of Linguistics, University of Texas at Austin, Austin, TX, United States; ^3^Department of Linguistics, Boston University, Boston, MA, United States

**Keywords:** English, French, clefts, contrast, interlocutors' expectations, existential inference

## Abstract

While much prior literature on the meaning of clefts—such as the English form “it is X who Z-ed”—concentrates on the nature and status of the exhaustivity inference (“nobody/nothing other than X Z”), we report on experiments examining the role of the doxastic status of alternatives on the naturalness of *c'est*-clefts in French and *it*-clefts in English. Specifically, we study the hypothesis that clefts indicate a conflict with a doxastic commitment held by some discourse participant. Results from naturalness tasks suggest that clefts are improved by a property we term “contrariness” (along the lines of Zimmermann, 2008). This property has a gradient effect on felicity judgments: the more strongly interlocutors appear committed to an apparently false notion, the better it is to repudiate them with a cleft.

## 1. Introduction

In many languages, a sentence expressing a single proposition can be cleft in twain, dividing the message over two clauses. Two examples are the English *it*-cleft (1-a) and its French counterpart the *c'est*-cleft (1-b).

**Table:** 

(1)	a. It's [David]_*F*_ who drank vodka.
	b. C'est [David]_*F*_ qui a bu de la vodka.

It is generally accepted that one purpose that clefting serves is to mark focus. Focus-marking entails that there are alternatives relevant for interpretation, and that those alternatives correspond to the focus-marked constituent (see e.g., Rooth, 1992; Krifka, 2008). In (1), the focus-marked constituent is the so-called *pivot* of the cleft, corresponding to the subject of the embedded clause in this case, and the alternatives correspond to other people who could have drank vodka, e.g., Paul, Jill, etc.

This paper investigates a relatively under-explored aspect of the focal alternatives determined by a cleft, namely their doxastic status for the interlocutor. In particular, we investigate the possibility that clefts signal a commitment on the part of the interlocutor to a proposition that conflicts with the one the cleft expresses, and that clefts serve to express opposition to that commitment. Using acceptability rating tasks, we provide experimental evidence that, *ceteris paribus*, both *it*-clefts and *c'est*-clefts improve in acceptability in proportion to the degree to which they indicate that an utterance runs contrary to a doxastic commitment on the part of the interlocutor (or another discourse participant).

Clefts have generally been analyzed as conveying three types of information (Halvorsen, 1978; Horn, 1981; Lambrecht, 1994), which we will refer to as the *Halvorsen components*. The first is the at-issue content, often referred to as the prejacent, which for a sentence of the form “it is X who Z-ed” is the proposition expressed by the canonical form “X Z-ed” (2-a). Second, clefts convey an existential inference, such that there exists an X who Z-ed (2-b). Unlike the prejacent, this aspect of clefts is typically taken to be a presupposition. Third, they convey an exhaustive inference such that X is the sole (or maximal) entity for which Z holds, e.g., (2-c)—the exact nature of which is still a matter of debate (see among others, Halvorsen, 1978; Atlas and Levinson, 1981; Wedgewood, 2007; Velleman et al., 2012; Büring and Kriz, 2013; Destruel et al., 2015). We briefly note for concreteness that we simply assume clefts have identical semantics as the prejacent, as argued for instance in Horn (1981)^1^.

**Table:** 

(2)	a. Prejacent: David drank vodka.
	b. Existential: Someone drank vodka.
	c. Exhaustivity: No one other than David drank vodka.

The doxastic status of these or other inferences for the hearer has typically not been discussed *p*er se, although Prince (1978) is one exception. On the basis of corpus evidence, she concluded that although *it*-clefts mark the existential as a “known fact,” yet “the information represented in *it*-cleft that-clauses does NOT have to be assumed to be in the hearer's mind.” Thus while the existential inference is presupposed, it can be what she terms an “informative presupposition.” She goes even further, claiming that “*it*-clefts make no assumptions about the hearer.” This latter claim is challenged by the data we present here.

Clefts have also been claimed to express *contrast* (Jespersen, 1927; Harries-Delisle, 1978; Sarnicola, 1988; Umbach, 2004; Patten, 2012). For English, this observation dates back to the work in which the term “cleft” was first coined; in perhaps the first general treatment of clefts, Jespersen (1927) claims “A cleaving of a sentence by means of *it is* (often followed by a relative pronoun or connective) serves to single out one particular element of the sentence and very often, by directing attention to it and bringing it, as it were, into focus, to mark a contrast (Jespersen, 1927, 147f.). For French, a similar observation is found in the seminal work of Lambrecht (1994), who argues that the *c'est*-cleft is the most natural way to signal *contrastive focus*, a type of focus that is sometimes distinguished from *information focus* (see e.g., Zimmermann and Onea, 2011). The former signals contrast, while the latter highlights new information.

How is contrast defined? On a broad view, adopted in the work of Vallduvi and Vilkuna (1998) (see also Selkirk, 2008; Lopez, 2009; Katz and Selkirk, 2011), a *kontrastive* expression *a* generates a membership set *M* = {…, *a*, …} which “becomes available to semantic computation as some sort of quantificational domain” (Vallduvi and Vilkuna, 1998). Contrast (formalized as *kontrast*) amounts to nothing more than membership in a salient set on this understanding. We note that this definition of contrast corresponds exactly to the definition of focus in Rooth (1985)'s Alternative Semantics in that the contrastive element generates a set of alternatives for the focused constituent.

Several more narrow conceptions of contrast exist as well. Rooth (1992) defines contrast as a subcase of a more general notion of focus; for him, a phrase α should be taken as contrasting with a phrase β if the ordinary semantic value of β is an element of the focus semantic value of α. É. Kiss (1998) writes that focus (for which she uses the term “identificational” focus) has the feature [+contrastive] “if it operates on a closed set of entities whose members are known to the participants of the discourse […]. In this case, the identification of a subset of the given set also identifies the contrasting complementary subset” (p. 267). This definition requires more than the broad one in that the set of alternatives to the focal element must also be *restricted* in size, and clearly *identifiable* by the discourse participants. Contrast has also been characterized with a requirement to exclude alternatives (Molnár, 2002; Kenesei, 2006); in other words, contrast entails *exhaustivity* on this view. Both Rooth's and Kiss's conceptions of contrast entail a requirement for a salient antecedent in the discourse, a requirement that goes beyond the three Halvorsen components ordinarily attributed to clefts.

There is some evidence that contrast in one of these narrower conceptions is indeed encoded by clefts, as they do appear to require a salient antecedent. For instance, while *it*-clefts often sound odd as direct answers to overt questions as in (3)—i.e., when there is no antecedent in the discourse—they are often much more natural as corrections, as in (4). In this case, the previous utterance being corrected provides exactly the kind of antecedent that Rooth mentions for a contrastive focus.

**Table:** 

(3)	A: Who cooked the beans?
	B: #It was John who cooked the beans^2^.

**Table:** 

(4)	A: I wonder why Alex cooked so much beans.
	B: Actually, it was John who cooked the beans.

Quantitative evidence that this contrast is robust comes from Destruel and Velleman (2014), who find that the context in 2 leads to the lowest naturalness ratings for clefts. If clefts encode contrast in Rooth's or Kiss's sense, then these differences can be explained^3^.

But even if the three Halvorsen components introduced in (2) are supplemented with a requirement for contrast in Rooth's or Kiss's sense (i.e., a requirement for the right sort of antecedent), the resulting theoretical picture still fails to capture certain facts about cleft behavior. In English, in contexts where an appropriate discourse-familiar alternative is indeed available, speakers may nevertheless choose *not* to use a cleft—its use sounding stilted and odd. Although experimental work on contrast in clefts is scarce, in a study conducted by Destruel and Velleman (2014), English speakers displayed a statistically robust preference for the canonical version in (5). They also rated the sentence in (5-b) as less natural than (6-b), despite the fact that (5-b) *does* have an antecedent available (viz. Canada), and (6-b) does not^4^.

**Table:** 

(5)	A: Darren sounded really excited about his vacation. I think he might be going to Canada.
	a. B: Actually, he's going to Mexico.
	b. B: ? Actually, it's Mexico that he's going to.

**Table:** 

(6)	A: We were planning Amy's surprise party for weeks. I can't believe she found out about it. Who told her about it?
	a. B: Ken told her about it.
	b. B: It was Ken who told her about it.

Finally, it is worth noting that there is evidence that in certain languages, clefts or other intuitive contrastive focus constructions do not always lead to the exclusion of alternatives; the strength of the exhaustive inference can in fact be modulated by the context. This has been argued, for instance, for clefts in St'át'imcets (Salish; Thoma, 2009) and French (Destruel and DeVeaugh-Geiss, 2018), for focus movement structures in K'ichee' which are arguably clefts (Mayan; Yasavul, 2013), and for non-cleft focus movement structures in Tangale (Chadic; Zimmermann, 2011) which, Zimmerman argues, still show signs of being contrastive in an important sense. Thus, if we want to retain the idea that clefts (and other focus movement constructions) are inherently contrastive, then these data suggest that defining contrast in terms of exclusion of alternatives may also miss the mark.

Given this backdrop, the question arises as to whether another factor might be relevant in better predicting the clefts' use in contrastive focus contexts, and more broadly, in characterizing the notion of contrast. We think that an interesting approach is found in Zimmermann (2008) and Zimmermann (2011), who proposes a definition calling on the notion of speaker-hearer *expectation*. This definition can therefore be thought of as *doxastic*. A focus constituent α is contrastive whenever the speaker assumes that “the hearer will not consider the content of α or the speech act containing α likely to be(come) common ground” (Zimmermann, 2008, 9). This suggestion is consonant with an earlier claim of Delin (1991), based on an extensive corpus study, that one of several different functions of *it*-clefts is “to correct some previous claim by challenging it.” Thus, our first research question is the following:

**Table:** 

(7)	**Research Question 1**
	What factor(s) other than the presence of a discourse-familiar alternative licenses clefts, and, specifically, does the attitude expressed toward salient alternatives affect the felicity of clefts?

Our research on this question builds on previous work by augmenting traditional analyses of contrast with what we term *contrariness*. In the spirit of Zimmermann (2008) and Zimmermann (2011), we take contrariness to relate to the degree of commitment that an addressee is established to have to a contrary focal alternative. More specifically, in our view, contrariness has the following three properties: (a) **contrast** (contrariness of one utterance in the discourse requires another utterance such that the first is an element of the alternative set of the second), (b) **contradiction** (taken together the two utterances are inconsistent, i.e., they entail falsity), (c) **strength** or degree of contrariness (which monotonically increases with the degree of commitment of the speaker to inconsistent propositions expressed by these utterances). We then distinguish between three imaginable hypotheses:

- The meaning components identified by Halvorsen (1978) (**the Halvorsen components**) are sufficient to capture the significance of a cleft construction. The contribution of alternatives to the meaning of a cleft lies solely in the exhaustivity component of the meaning.
- In addition to the Halvorsen components, clefts signal a **non-doxastic** type of contrast, of the type characterized by É. Kiss (1998) or Rooth (1992), incorporating a requirement for an appropriate antecedent.
- In addition to the Halvorsen components, clefts signal a **doxastic** type of contrast (i.e., *contrariness*). The nature of the clefted alternatives involves a contrast between interlocutors' expectations^5^.

The experiments reported in this paper set out to test Hypothesis (iii)—we hypothesize that in addition to the core components in (2), clefts incorporate a requirement that the ordinary meaning is contrary to a previously salient focal alternative. Put slightly differently, we expect clefts to be optimal candidates in contexts where they do more than just introduce a linguistic contrast, but rather are used as a response to an (explicit) contrary claim. We expect this effect to be gradient on felicity judgments: the more strongly interlocutors appear committed to an apparently false notion, the better it is to repudiate them with a cleft. Crucially, this doxastic definition allows for *degrees* of contrast, corresponding to stronger or weaker conflict with expectations, and we argue that these degrees correlate with clefts' naturalness. On this basis, the slight infelicity of (5-b) might be explained as follows: Although there is some contrast between B's claim and what A has stated previously, A's hedging (“I think he might…”) indicates only a mild commitment to a contrary proposition, and this mild commitment to a contrary proposition does not suffice to make a cleft fully felicitous for B. Compare this with the much more strident rebuttal of what the hearer suggests is some people's view found in this naturally occurring example cited by Hedberg (1990)^6^:

**Table:** 

(8)	JM: Some people think that Reagan's administration is at its LOWEST ebb, its NADIR. Do you agree, Eleanor?
	EC: Absolutely not. The Reagan-Baker Administration is in FINE shape. It's the BUCHANAN administration that's having PROBLEMS.

A second issue central to our current research concerns the grammatical reflex of contrast across languages. Indeed, while the bulk of the past theoretical literature on focus and clefts has been developed around (introspective judgments for) English, cross-linguistic counterparts to the *it*-cleft are also noted to express contrast, such as the French *c'est*-cleft, as mentioned earlier. But, as Repp (2016) notes, languages might differ with respect to the grammatical sensitivity they have to particular aspects of the (set of) alternatives. The author says that “for instance, the view that alternativeness equals contrastiveness might make the right prediction for the application of particular strategies in language *x* whereas in language *y* similar marking strategies might require the presence of a clearly identifiable alternative set." This seems particularly relevant when comparing clefts in languages like French and English since, while both it- and *c'est-*clefts can express contrast, there are subtle and crucial differences in their distribution. First, the French cleft is used more commonly than its English counterpart (Carter-Thomas, 2009), in particular in comparison to canonical sentence forms (SVO). The reason appears to be primarily prosodic: whereas English can shift prosodic prominence to match the location of the focus constituent, French is more rigid, and prosodic stress is required to appear at the right edge of an intonation phrase. The *c'est*-cleft, despite adding syntactic complexity, circumvents this prosodic restriction by creating an extra intonational boundary that can align with the focus constituent (Hamlaoui, 2008). Consequently, the *c'est*-cleft constitutes the default strategy to signal focus (also known as *information* focus), especially on grammatical subjects (Lambrecht, 1994; Destruel, 2013; Féry, 2013). Second, the French *c'est*-cleft can be used in focus contexts where the English cleft is prohibited; for instance to signal focus on the entire sentence rather than on a single element (i.e., *broad* focus). Given that clefts have a broader distribution in French than English, our paper seeks to address a second research question:

**Table:** 

(9)	**Research Question 2**
	Does dependency of the status of alternatives differ between these two languages?

Our research on the second question builds on prior work by directly comparing the role of contrariness in two languages that have different use-conditions for the cleft construction. Given the subtle differences in clefts' use in French and English, we expect that the two languages may differ as to how contrastive a discourse must be before the cleft is considered most natural.

To the best of our knowledge, there have been very few attempts to investigate the contrastive aspect of clefts experimentally (but see Destruel and Velleman, 2014), and especially across languages that differ in their use of clefting as a strategy to mark focus. Moreover, in attempts that do exist, contrast is not often operationalized in a gradient way, i.e., studies typically compare highly contrastive contexts to non-contrastive ones, leaving aside the potential different degrees that contrast can have. Given these observations and the background information presented thus far, this paper aims to bridge the theoretical and the empirical literature on contrast in clefts. The remainder of the paper is structured as follows: We present the studies in section 2, discuss their results in light of current views of contrast and correctivity, and clefts' meaning in section 3. We end with concluding remarks as well as avenues for future work in section 4.

## 2. The Studies

Recall that the paper examines two research questions, repeated in (10) and (11) for convenience.

**Table:** 

(10)	**Research Question 1**
	What factor(s) other than the presence of a discourse-familiar alternative licenses clefts, and, specifically, does the attitude expressed toward salient alternatives affect the felicity of clefts?

**Table:** 

(11)	**Research Question 2**
	Given that clefts have a broader distribution in French than in English, does dependency of the status of alternatives differ between these two languages?

Our investigation includes three tasks conducted in English and French. Two pre-tests were designed to provide baseline ratings for the existential inference in target sentences and for the strength of commitment of Speaker A in the context, respectively; the main task consisted of naturalness ratings for clefts and canonical sentences in six contexts that instantiated different degrees of contrariness. The experimental stimuli for these three tasks were always presented in written form and were based off of the same source sentences, which were translated by a French native speaker for the French version of the experiment. What differed across tasks regarding the materials was which part of the stimuli participants got to see and judge. Given this, we present the common elements of the three tasks in section 2.1. We present the details for each task—i.e., design, procedure and results—in sections 2.2–2.4.

### 2.1. Methods

#### 2.1.1. Materials

The experimental stimuli consisted of short dialogues between two speakers. All dialogues included a **background** (Speaker A) as in (12), and a **comment** (Speaker B) presented either in a canonical SVO or in a cleft form, as in (13). Note that the sample stimuli in (12)–(13) illustrate the condition in which the focus is on the grammatical subject. See (14) for an example of the object condition, and Appendix A for a larger sample of stimuli. The background always contained three sentences. The first two established the story and the last one contained the information on which B's comment was based. The last sentence in Speaker A's part was crucial in our experiment; this is the sentence we modulated to create six contexts with varying degrees of contrariness, illustrated in (12-a)-(12-f). These six contexts varied according to four factors: Grammatical Function, Contradiction, Commitment and At-issueness. We detail them individually hereafter. For each of the six contexts, we created 12 lexicalizations, so 72 experimental dialogues per grammatical function or 144 in total, and this for each language.

**Table:** 

(12)	Speaker A: We were planning Amy's surprise party for weeks. I can't believe she found out about it. […]
	a. Non-contradictory, At-issue (no contr.)…I guess someone from the staff told her.
	b. Weak, At-issue (weak) …I guess Alice must have told her.
	c. Weak, Non-At-issue (weak nai) …And Alice—who I think, probably went and told her about it—just laughed and said it was no big deal!
	d. Strong, At-issue (strong) …Alice told her about it, you know.
	e. Strong, Non-At-issue (strong nai) …And Alice—who went and told her about it—just laughed and said it was no big deal!
	f. Strong Presuppositional, Non-At-Issue (strong pre.) …I'm annoyed that Alice told her about it!

**Table:** 

(13)	Speaker B: Yeah/ Actually, […]
	a. …Ken told her about it. *(canonical form)*
	b. …it's Ken who told her about it. *(cleft form)*

The first factor varied was grammatical function of the focused element, that is whether the element that B commented on was the grammatical subject or the object. Example (14) illustrates the object condition for context no contr.

**Table:** 

(14)	Object condition, no contr.:
	a. Speaker A: Look at John this evening! He's all dressed up. […] I guess he's going out with someone from the marketing team.
	b. Speaker B: Yeah, he's going out with Karen/ Yeah, it's Karen he's going out with.

The second factor, contradiction, refers to whether or not the information in Speaker B's comment contradicted the information stated in the last sentence uttered by Speaker A. We can think of this variable as binary: The first context we designed (no contr.) has a contradiction value of 0 (i.e., it is non-contradictory) because there is no other identifiable salient individual in A's part. The other five contexts have a contradiction value equal to 1; they are contradictory in the sense that there is one alternative explicitly given in the discourse, thus being clearly identified. In the non-contradictory context, B's comment was always introduced by “Yeah/*Ouai*,…,” while in all others, B's comment was introduced by “Actually/*En fait*,…”.

The third factor we manipulated was at-issueness, which refers to whether or not the relevant proposition in A's speech commented on by B was at-issue. The motivation behind including at-issueness as a factor comes from Destruel and Velleman (2014), who also argue for the relevance of *contrast in expectation* in the interpretation of clefts, and propose that two types of expectations may be at play; not just expectations about the state of the world but also expectations about the shape and direction of discourse. The latter type is directly relevant here since it may involve beliefs about the direction in which the discourse is going, expressed, among other ways, by marking content as at-issue or not-at-issue. We assume that interlocutors taking part in a discourse will generally address the propositions that are currently at-issue.

Finally, we varied commitment, which corresponds to the strength with which Speaker A is committed to their statement. Expanding on prior studies on the (grammatical) reflexes of contrast, we take this factor to be gradient; it can vary in strength depending on how the speaker chooses to express their beliefs. We designed contexts that varied in ways that we assumed would affect the level of commitment^7^. We used a variety of attitude verbs and adverbs to encode these various degrees. For instance, in the weak and strong conditions (contexts weak to strong nai), the speaker respectively expresses a low or a high degree of commitment toward the asserted prejacent proposition. In context strong pre., on the contrary, the prejacent is presupposed; the speaker expresses a personal, subjective opinion about the truth of another asserted proposition in the sentence (i.e., “I'm annoyed that Alice told her about it!,” in (12-f)). Since at-issueness reflects differences in whether a speaker has decided to foreground commitment to a proposition, we anticipated that at-issueness might affect the perceived level of commitment. Further, different types of non-at-issue material (conventional implicatures vs. presuppositions) might also be expected to affect perceived commitment in different ways, e.g., because presupposed non-at-issue material is often taken to reflect a shared commitment, whereas other types of non-at-issue material, such as conventional implicatures from parenthetical, are not. Therefore we included both stimuli in which the target proposition was presupposed, and material in which it was conventionally implicated (in the sense of Potts, 2005).

A pre-test (task 2), which we detail in section 2.3 was conducted prior to the main task in order to assess whether our contexts were indeed different with respect to the strength of commitment encoded, as we conceived them to be. In our view, more strongly expressed commitment lead to stronger conflict between interlocutors, and thus we hypothesized that clefts are more natural in cases when the level of conflict between interlocutors is maximal, or in other words, when clefts are used as responses to an (explicit) contrary claim.

We now turn to discussing each task individually, the two pre-tests first (sections 2.2 and 2.3) and then the main task (section 2.4).

### 2.2. Task 1: Strength of Existential Inference

#### 2.2.1. Participants

We note that all participants in Task 1 were different from the participants who completed Task 2 and the main task.

For English: We recruited a total of 65 participants (all undergraduates at a midwestern university, ages: 19–23; median: 20) from a first-year language class. Subjects were given extra-credit for their participation and were all naive as to the goal of the experiment.

For French: We recruited 48 monolingual native speakers of French. All were given monetary compensation for their participation and were naive as to the goal of the experiment. Participants were from the regions of Pau, Toulouse and Albi in Southwestern France. Overall, 61% were undergraduate students, 34% graduate students, and 5% staff working at the university.

#### 2.2.2. Design & Procedure

The goal of this first test was to measure the strength of the existential inference in Speaker A's part, i.e., **how likely is it that A believes someone “Z-ed”?** This is necessary to ensure that any effect of contrariness we find is not an artifact of variation among items with respect to the strength of the existential inference that they give rise to. The test was delivered via the web-based survey site Qualtrics. Participants sat in front of a computer screen and read a total of 24 backgrounds (A's part), pseudo-randomized among 24 fillers (recall that participants only saw and rated Speaker A's part of the dialogue in this task.) On each trial, after reading A's part, participants were asked to judge, on a scale from 1 to 7, how likely is it that A thinks that someone Z-ed. So for instance, given no contr. context in (12-a) above, participants were asked how likely is it that “A believes someone told Amy about her surprise party” (1 corresponding to extremely unlikely and 7 to extremely likely). The procedure for English and French was exactly similar; French speakers provided judgments based on the question “*Quelle est la probabilité que A pense que quelqu'un a Z?*”

#### 2.2.3. Results

Mean probability ratings for the strength of the existential inference in A's part are presented in Table 1, for English and French.

**Table 1:** Mean probability judgments for pre-test 1 (Strength of existential inference).

	**Mean ratings (subjects)**		**Mean ratings (objects)**		**Overall ratings**	
	**English**	**French**	**English**	**French**	**English**	**French**
No contr.	4.6	4.5	4.4	4.8	4.5	4.65
Weak	6.5	6.3	6.3	6.4	6.4	6.35
Weak nai	6.5	6.4	6.5	6.4	6.5	6.4
Strong	6.6	6.4	6.7	6.6	6.7	6.5
Strong nai	6.4	6.8	6.4	6.6	6.4	6.7
Strong pre.	6.7	6.7	6.7	6.8	6.7	6.75

Visual inspection of these averages suggests that participants deem the likelihood of speaker thinking that someone Z-ed lower for the context that lacks a contrast between A's sentence and B's response (i.e., context no contr., μ = 4.5/4.65), vs. other contexts (where μ is consistently above 6.3)—and this quite similarly in both languages.

To determine whether participants' existential ratings varied depending on the fixed-effect predictor contrast (sum-coded prior to analysis as -1/1 for context (no contr. vs. others, respectively), we fit a linear mixed effect model to the data for each language. The two models included the maximal random effects structure justified by the data: random by-item intercepts, random by-participant intercepts and random slopes for contrast by item and participant. *P*-values were obtained by likelihood ratio test of the full model with the effect in question against the model without the effect in question. Results reveal a significant effect of contrast both in English (β = 2.043, *SE* = 0.091, *t* = 22.24, *p* < .001), and French (β = 1.62, *SE* = 0.24, *t* = 6.72, *p* < .001) suggesting that, as expected, there was a difference in ratings between the non-contrastive context (#1, in (12-a)) vs. the others where a conjecture was present [contexts in (12-b)–(12-f)].

Crucially though, when looking only at the contradictory contexts, we see that the ratings do not significantly differ from each other with respect to A's commitment to existence. This is an important finding since it indicates homogeneity across these contexts. If we also find that these contexts differ in the strength of A's commitment to a statement that B will contradict (as they were designed to do and is tested in task 2), then we will be able to test our prediction that clefts' naturalness is best predicted by a doxastic contrast (i.e., Hypothesis iii.).

### 2.3. Task 2: Strength of Commitment

#### 2.3.1. Participants

We note that all participants in Task 2 were different from the participants who completed Task 1 and the main task.

For English: We recruited a total of 65 participants (all undergraduates at a midwestern university, ages: 18–21; median: 20) from a first-year language class. Subjects were given extra-credit for their participation and were all naive as to the goal of the experiment.

For French: We recruited 48 monolingual native speakers of French. All were given monetary compensation for their participation and were naive as to the goal of the experiment. Participants were from the regions of Pau, Toulouse and Albi in Southwestern France. Overall, 83% were undergraduate students, 15% graduate students, and 2% staff working at the university.

#### 2.3.2. Design & Procedure

Recall that the different contexts in our study were designed to reflect the idea that contrast is not simply a binary notion, but rather that speakers' beliefs are gradient. We created four levels—*non-contradictory, weak, strong* and *presuppositional*—with the underlying assumption being that commitment would get increasingly stronger across these levels. The present task was conducted to test precisely this assumption, that is to directly measure **how strongly is A committed to “X Z-ed.”** Thus, subjects who took part in this task only saw and rated Speaker A's part of the dialogue. The test was delivered via the web-based survey site Qualtrics. Participants sat in front of a computer screen and read a total of 24 contexts (A's part) pseudo-randomized among 24 fillers. On each trial, after reading A's context, they were asked to judge, on a scale from 1 to 7, how strongly is A committed to the fact that X Z-ed. So for instance, given context no contr. in (12-a) above, participants were asked how strongly is Speaker A committed to the fact that “someone from the staff told Amy about her surprise party” (with 1 corresponding to extremely uncommitted and 7 to extremely committed). Here again, the procedure for English and French was exactly similar; French speakers provided judgments based on the question “*À quel point est-ce que A pense que X a Z?*”

#### 2.3.3. Results

Results for both languages are reported in Table 2. Looking at the ratings descriptively, we indeed observe a strengthening trend across contexts. We see that context no contr. is given the lowest commitment scores of all contexts, and that contexts strong, strong nai and strong pre.—which were designed to contain a stronger commitment of A to the prejacent proposition—are indeed being rated higher than contexts weak and weak nai, which were meant to weakly commit A to the prejacent. Interestingly, we do not see a major difference between the strong and the presuppositional context.

**Table 2:** Mean commitment judgments for pre-test 2.

	**Mean ratings (subjects)**		**Mean ratings (objects)**		**Overall ratings**	
	**English**	**French**	**English**	**French**	**English**	**French**
No contr.	2.2	2.3	2	2.1	2.1	2.2
Weak	3.6	3.8	3.9	4.1	3.8	4
Weak nai	2.7	3	2.6	3.5	2.7	3.2
Strong	6.1	6.4	6.1	6.1	6.1	6.25
Strong nai	5.5	5.8	5.3	5.8	5.4	5.8
Strong pre.	5.3	6	5.6	6.2	5.5	6.1

Statistically, we fit a linear mixed effect model to the data for each language to determine whether participants' judgments varied depending on the fixed-effect predictor commitment. We were most interested in the following comparisons: comparing the context with no contradiction (no contr.) to context with weak at-issue commitment (weak context), and comparing weak contexts (weak and weak nai) to strong contexts (strong and strong nai). We used sum-coding prior to analysis (i.e., -1/1) for each level in each comparison. The models included the maximal random effects structure justified by the data: random by-item intercepts, random by-participant intercepts and random slopes for commitment by item and participant. *P*-values were obtained by likelihood ratio test of the full model with the effect in question against the model without the effect in question. Concentrating on the comparison between our no contr. and weak contexts, we found a significant effect of commitment both in English (β = 1.54, *SE* = 0.017, *t* = 3.31, *p* < .001), and French (β = 1.47, *SE* = 0.11, *t* = 3.56, *p* < .001). We also found a significant effect of commitment when comparing weak contexts to strong ones, both in English (β = 2.29, *SE* = 0.29, *t* = 5.24, *p* < .001), and French (β = 2.11, *SE* = 0.025, *t* = 4.98, *p* < .001). Overall, these results are welcome since they suggest that the contexts we designed *did* differ in the strength of A's commitment to a statement that B will contradict to the prejacent, and this for both languages. We can now turn to the main task, testing Hypothesis (iii).

### 2.4. Main Task

#### 2.4.1. Participants

We note that all participants in the main task were different from the participants who completed Task 1 and the Task 2.

For English: We recruited 64 participants on Amazon's Mechanical Turk with U.S. IP addresses (ages: 20–61; median: 36). They were paid $1 for their participation. Subjects who did not self-identify as native English speakers were not considered.

For French: We recruited 48 monolingual native speakers of French. All were given monetary compensation for their participation and were naive as to the goal of the experiment. Participants were from the regions of Pau, Toulouse and Albi in Southwestern France. Overall, 77% were undergraduate students, 17% graduate students, and 6% staff working at the university.

#### 2.4.2. Design & Procedure

On each trial of this task, participants saw the whole dialogue, that is, A's background followed by Speaker B's comment (appearing either in cleft or canonical form). They were asked to judge the naturalness of B's sentence given A's on a seven-point Likert scale, with 1 corresponding to extremely unnatural and 7 to extremely natural.

We tested the effect of four factors on participants' ratings of cleft and canonical sentences: (i) existence (based on measures collected in task 1), (ii) grammatical function (subject vs. object), (iii) at-issueness, and (iv) contrariness. The factor contrariness was operationalized as the product of contradiction and strength of commitment (Contrariness = Contradiction ^*^ Strength of Commitment). Contradiction, as mentioned in section 2.1.1, is either equal to 0 in the non-contradictory context (context no contr. where Speaker B does not say anything that conflicts with what Speaker A says) or equal to 1 in the others. Consequently, items in the non-contradictory context had a contrariness value of 0. Items in contradictory contexts (contexts weak to strong pre.) had a contrariness value equal to 1 (their contradiction value) * the value of Speaker A's commitment to the conflicting proposition, as measured in task 2.

If the data supports Hypothesis (iii), we expect to find that clefts are rated as more natural in the contexts where the level of contrariness is higher. We counterbalanced the experimental dialogues across 12 lists so that each participant judged a total 24 items (12 subjects and 12 objects). The order of the items was pseudo-randomized among 24 fillers.

#### 2.4.3. Results

**Figure 1:**
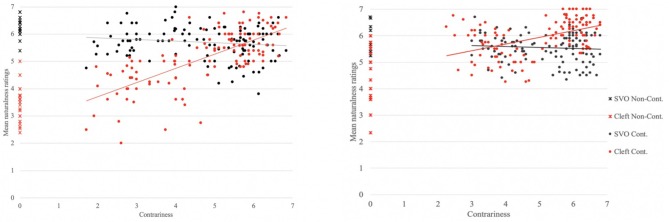
Naturalness ratings for English **(Left)** and French **(Right)**.

In the following, we begin by assessing our results descriptively, then we turn to the statistical analyses. Results combined for both sentence forms (clefts and SVO canonical sentences) and collapsed for grammatical function (subjects and objects) are illustrated in Figure 1, for English on the left panel and French on right panel. On Figure 1, red-colored markers represent clefts and black-colored markers represent canonical sentences. The cross-shaped markers on the y-axis indicate the naturalness ratings in the non-contradictory context (no contr.). The circle-shaped markers indicate the ratings for the other contexts. Moreover, we note that the labels on the x-axis do not correspond to the number of our contexts, bur rather encode the contrariness values attributed to items in these contexts on a 7-point Likert scale, as per the results we gained in task 2 (discussed in section 2.3). Put simply, our x-axis represents the product of contradiction and commitment (as measured in task 2). Tables that include the mean naturalness ratings for each of our six contexts, per language and sentence form, can be found in Appendix B.

Inspecting the data for English, the figure reveals that the ratings for the cleft seem the most affected by contrariness, displaying the steepest increase across language and conditions (as illustrated by the upward trend in the position of the red dots). Indeed, clefts' ratings were the lowest of all in the no contrno contr. context (μ = 3.39), but increased as contrariness intensified (μ = 5.9 in strong pre. context). The picture is quite different for canonical sentences: They were rated as very natural in the non-contradictory context (μ = 6.25), which should come to no surprise since in English, canonical sentences constitute an unmarked sentence form and are commonly used to answer an explicit wh-question. Interestingly, their felicity did not improve much with contrariness, but in fact slightly decreased (μ = 5.6). Despite this decrease though, canonical sentences were never judged infelicitous (in the sense of being below the midpoint of the 7 point scale), and were only slightly worse than clefts in the strong pre. context.

Turning to French, we also observe an increase in clefts' naturalness as contrariness gets stronger, but to a much lower degree than in English. This is mainly due to the fact that French clefts are already rated fairly high in no contr. context (μ = 4.56), as opposed to the English clefts (μ = 3.39), which is expected given that clefts are the most natural way to signal focus in the former language, especially with grammatical subjects (as argued by Lambrecht, 1994 among others, and empirically substantiated in Destruel, 2013; Féry, 2013). Similarly to English though, canonical sentences behave differently from clefts: While being rated highly in non-contradictory contexts (μ = 5.64), their naturalness does not improve as the level of contrariness rises (μ = 5.03). The first part of this result is interesting because it is at odds with many past accounts in the French literature that claim canonical sentences are highly disprefered in focus contexts (Lambrecht, 1994; Katz, 1997; Doetjes et al., 2004). What could be happening is that canonical sentences are rated as more felicitous in our study because they appear in written form, rather than in colloquial speech. We return to this point in the general discussion in section 3.

**Figure 2:**
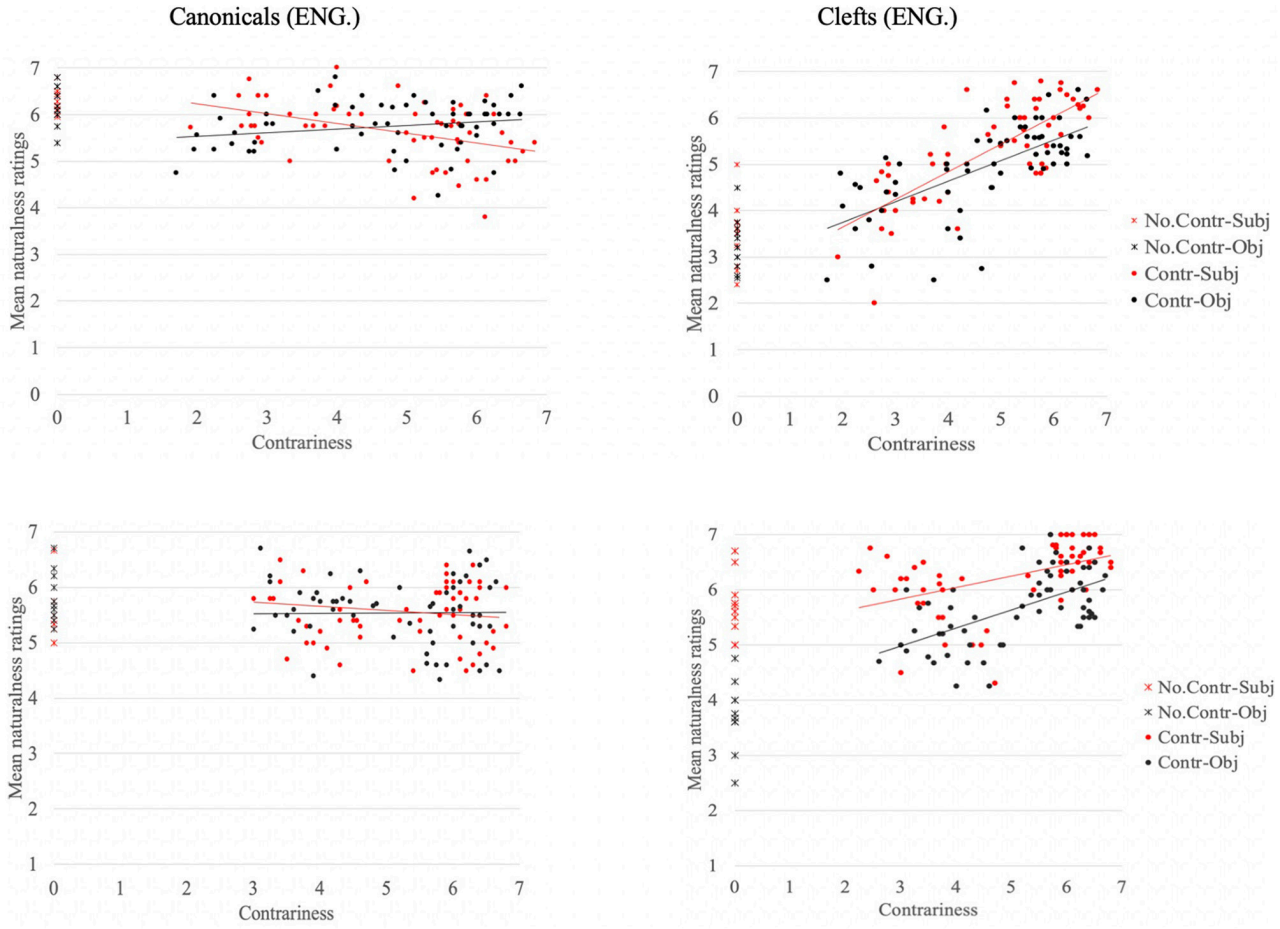
Naturalness ratings per grammatical function, for canonical sentences **(Left)** and clefts **(Right)** in English **(Top)** and French **(Bottom)**.

Now, we explore the data by grammatical function (subjects vs. objects), as illustrated in Figure 2, where ratings for canonical sentences appearing in the left panels and ratings for clefts appear in the right panels. The data for English are on the top two graphs; the data for French are at the bottom. On all plots, the red-colored markers represent the subject condition and black-colored markers represent the object condition. The cross-shaped markers represent the data for the non-contradictory context, and the circle-shaped markers represent the data for the contradictory contexts.

First, we concentrate on the right panels—the results for cleft sentences. Visual inspection of Figure 2 reveals an asymmetry in clefts' ratings for French in the non-contradictory context (bottom right graph): Object clefts (black circles) appear clearly lower than subject clefts (red circles) (μ = 3.68 vs. μ = 5.43, respectively). This asymmetry relating to argument hierarchy is in line with the past literature and recent empirical evidence that suggest subject focus obligatorily induces a non-canonical structure while object focus only optionally does so since objects appear by default rightward, where prominence is assigned in French (Lambrecht, 2001; Destruel, 2016). We note that evidence for such an asymmetry is also provided cross-linguistically in languages such as Spanish (Buring and Gutierrez-Bravo, 2001), Northern Sotho (Zerbian, 2007), Georgian and Hungarian (Skopeteas and Fanselow, 2010b). This asymmetry is absent from our English data (see top right graph, μ = 3.26 and μ = 3.52 for objects vs. subjects, respectively), which is in line with the English results from an elicitation task reported in Skopeteas and Fanselow (2010b).

Second, looking at the data for canonical sentences, we see no such asymmetry in either of the two languages. Canonical sentences are rated equally high whether focus appears on the subject or the object, especially in the no contr. context (English: μ = 6.3 for subjects and μ = 6.2 for objects; French: μ = 5.8 for subjects and μ = 6 for objects). Here as well, we note that the results for French are at odds with Lambrecht's claim that canonical sentences with lexical subjects is not the predominant pattern that surfaces in the spoken language (Lambrecht, 1987).

We conclude by reporting on the statistical analyses. We conducted mixed-effects linear regressions predicting clefts and canonical sentences' naturalness ratings in English and French from fixed effects of interest (i.e., grammatical function, at-issueness, existence and contrariness), and the following random-effect structure: random intercepts and slopes for the fixed effects of interest, and their interaction when relevant, per participant and item. When the maximal models did not converge with the maximal random effects structure, they were re-conducted with the next maximal random effects structure until convergence was achieved. All fixed effects were centered before entering the analysis. To assess whether inclusion of a given factor significantly improved the fit of the overall model, likelihood-ratio tests were performed that compared two minimally different models, one with the fixed effects factor in question and one without, while keeping the random effects structure identical (Barr et al., 2013). The full model had the following structure: (Ratings ~ 1 + At-Iss * GramFunct * Exist * Contrariness + Maximal RES). In the following, we report on estimates, standard errors, and *t*-values for all models (with any *t*-value exceeding 1.96 considered statistically significant with *p* < 0.05), as well as the χ^2^ and *P*-values from the likelihood-ratio tests. Results were obtained using the *lmer* function of the *lme4* package (GPL-2|GPL-3, v.1.1-13; Bates et al., 2015) in the R environment (GPL-2|GPL-3, v.3.3.3; R Core Team, 2017).

We split the data prior to analysis, first looking at ratings for clefts in English. Within this data set, we found no effect of at-issueness (β = -0.16, *SE* = 0.11, *t* = -1.44), suggesting that when clefts were used to signal an unexpected discourse move (i.e., to signal contrast on an element that was part of the non-at-issue content of A's speech) they were not drastically better than when commenting on an at-issue part of discourse. There was also no effect of grammatical function (β = 0.31, *SE* = 0.09, *t* = 1.13), such that the ratings for subject and object clefts were not significantly different. There was, however, an effect of existence (β = 0.52, *SE* = 0.08, *t* = 6.55), suggesting that clefts were rated significantly better in contexts where the existence of the element to be contrasted is assumed. Of the three nested models, the one that gave the best fit to the data was the model that simply included the factor existence (χ^2^ = 9.72, *p* < 0.01). Of most interest to us, the factor that had the largest effect on clefts' ratings was contrariness (β = 0.01, *SE* = 0.001, *t* = 11.06)—the model that included this factor gave a significantly better fit to the data compared to the model that did not (χ^2^ = 77.85, *p* < 0.01). This supports our hypothesis (Hypothesis iii) that clefts' naturalness is affected by the degree to which a speaker is committed to a (false) claim.

The picture is similar for French clefts in that at-issueness had no effect either (β = -0.01, *SE* = 0.08, *t* = -1.34), but existence did (β = 0.64, *SE* = 0.05, *t* = 12.03). One notable difference is that there was an effect of grammatical function (β = 0.88, *SE* = 0.07, *t* = 11.84), suggesting that subject clefts were given significantly better ratings than objects clefts. This result is unsurprising given what we already mentioned; that clefts are argued to be the default strategy to signal subject focus in French. The factor contrariness, although to a lesser extent than in English, also had a significant effect in predicting clefts' naturalness (β = 0.008, *SE* = 0.001, *t* = 5.55); a model that included this factor gave a better fit to the data than a model without it (χ^2^ = 41.18, *p* < 0.01).

Finally, we report on the naturalness rating for the data set of ratings for canonical sentences. In English, we only found an effect of contrariness (β = -0.092, *SE* = 0.017, *t* = -5.24); all other factors did not significantly affect the felicity of canonical sentences. In French, we found no effect of at-issueness (β = 0.11, *SE* = 0.10, *t* = 1.06) or of existence (β = 0.09, *SE* = 0.08, *t* = 1.02), but there were an effect of grammatical function (β = -0.20, *SE* = 0.09, *t* = -2.25) and of contrariness (β = -0.007, *SE* = 0.001, *t* = -4.92).

## 3. General Discussion

Clefts have long been noted to be focus-marking devices, often expressing a more special type of focus, i.e., contrastive focus, as opposed to a “simpler” type of focus generally referred to as *informational* focus (É. Kiss, 1998). Yet, traditional definitions of contrast appear unable to fully predict when these structures are most felicitous. This observation constituted the core motivation for our studies—our goal being to explore the relationship between the rhetorical role of focal alternatives and the naturalness of clefts in French and English, as per the two research questions in (10) and (11). More specifically, the experiments were designed to test the idea that clefts incorporate a requirement that the ordinary meaning is contrary to a previously salient focal alternative, which we operationalized via the notion of *contrariness* (i.e., strength of commitment * contradiction).

In the following, we first summarize the main experimental results and how they speak to our research questions, then we turn to discussing the implications of our findings for accounts on the meaning of clefts, definitions of contrast, and theories of focus.

Regarding the first research question, the experiment provided evidence that, although the presence of a focal alternative in the discourse context does increase the naturalness of clefts, it does not suffice to explain when clefts are preferred. In fact, while controlling for other factors known to influence the acceptability of clefts, naturalness ratings were significantly impacted when a doxastic contrast was involved: clefts are better in contexts where they indicate that an utterance runs contrary to a doxastic commitment held by the hearer, and the results are consistent with there being a requirement for a salient contrary doxastic commitment, whether that of an addressee or some other individual. We also found that whether contrastive content was marked as being at-issue or not did not significantly affect clefts' naturalness. This suggests that metalinguistic expectations about how a contrary point of view is changing in the discourse are less relevant to the acceptability of clefts than are salient beliefs about the world.

Our second research question asked whether dependency on the status of alternatives differs between French and English. In considering this question, it is necessary first to tease apart what we take to be independent differences between the two languages. Specifically, we need to separate the effects of grammatical function from the effects of the status of alternatives. Our experiments showed, in agreement with past literature, that in French but not English there is an effect of grammatical function: whereas in French subjects are more naturally clefted than objects, this is not the case for English. Our statistical analysis shows that once we control for this cross-cutting factor, we can see that clefts in the two languages exhibit very similar dependencies on the status of alternatives. In both languages clefts are more natural when there is doxastic contrast.

Even though our study was designed primarily to examine the use of clefts, another way to look at the data is to examine what happens in comparison with canonical sentences. It is often thought that their use is correlated: Lambrecht (1994) has claimed that clefts in French are used when canonical sentences are infelicitous. We find qualified support for this hypothesis, and indeed the effects are found in both languages we studied. On the one hand, canonical sentences were never rated as being highly infelicitous in our study. This fact appears to partially undercut Lambrecht's claim, since he motivated it on the basis of judgment and observational data suggesting that canonical sentences in French with lexical (i.e., non-pronominal) subjects are infelicitous. To the extent that we can operationalize infelicity as corresponding to mean ratings in the lower half of our 7 point scale, this is not what we found. While the results on clefts in non-contrastive conditions showed that the French speakers in our sample were prepared to mark at least some sentence types as being infelicitous in some conditions, they never rated canonical sentences as infelicitous. Thus, if French speakers only used clefts when their canonical counterparts were strictly infelicitous, they would be predicted to never use clefts at all, or at least not in any of the conditions we tested. Nonetheless, we did find reduced acceptability for canonical sentences in some conditions, specifically for sentences in French in which the context might lead to an expectation of focus on the subject, and for canonical sentences in both French and English for which the context led to a high level of contrariness. It is precisely in these conditions that cleft sentences have their highest mean acceptability in our study. Hence there is, at the very least, a correlation: the less acceptable canonical sentences are in a given context, the more acceptable corresponding cleft sentences are in that same context. It is thus plausible that at least one of the factors motivating cleft use is dispreference for use of the canonical form, albeit that it would be far too strong to say that cleft sentences are used when the canonical counterpart is unavailable.

What are we to make of the fact that canonical sentences in both French and English were judged to be slightly, but significantly degraded in contexts imposing a high degree of contrariness? One hypothesis consistent with this result is that the grammar directly imposes a penalty on the use of canonical sentences in such contexts. However, here, the style of Lambrecht's analysis provides an alternative way to look at the data. Lambrecht's model is paradigmatic, i.e., based on the contention that language users consider competing forms, and that suitability of one form depends on the availability and appropriateness of competing forms. It is consistent with the data that while the canonical form is unmarked, and has no requirements on (non-)contrariness, the cleft construction is a marked form which is specifically used when the meaning is also marked, for example in terms of contrariness. Thus in these situations, following what Horn (1984) called the division of pragmatic labor, the marked form is expected to be used in the marked context, and the unmarked form is then pragmatically dispreferred in these contexts. This type of explanation of the observed degradedness of canonical sentences in some contexts provides broad support for a Lambrechtian approach, even if his specific claims appear overly strong. Of course, it is also compatible with our data that cleft sentences are unmarked, and involve no inherent, conventionalized contrariness preference, but that canonical sentences have a conventional preference for non-contrary contexts. This seems a *prima facie* implausible analysis, reflected in the fact that the linguistic convention of terming the SVO form in English and French “canonical” already suggests that it is the unmarked form. We merely note that our data does not mitigate strongly against such an analysis.

As discussed in the introduction, the past literature on the meaning of clefts has largely characterized clefts as having three meaning components, cited in (2). Furthermore, much work has concentrated on describing the nature of exhaustivity, arguing either that it is semantically encoded in the cleft itself (Atlas and Levinson, 1981; Percus, 1997; É. Kiss, 1998; Hedberg, 2013), or that it arises as a result of pragmatic reasoning on the discourse context (Horn, 1981). In general, it is often supposed that aspects of meaning which are “baked” into the conventional meaning of an expression should surface more robustly than aspects of meaning and use which are derived indirectly, and involve pragmatic reasoning. Based on this premise, prior experimental research (Byram-Washburn et al., 2013; Destruel, 2013) has suggested that exhaustivity is pragmatic. The pattern of data that we have reported on in the current paper might then also be taken to suggest that contrariness requirements are derived via some pragmatic process, since, our contrariness data resemble prior exhaustivity data in that we observed gradient differences in judgments across conditions, rather than clear categorical effects with sharp boundaries between felicitous and infelicitous uses of clefts. However, we must note here that absent more constraints on possible conventional theories and the way they relate to judgment data, such a conclusion would be premature.

To see how our data might in principle be modeled in terms of linguistic conventions, let us briefly describe one such model. Call a base grammar one in which there is a certain set of requirements on the epistemic attitude of a salient individual toward a contrary proposition to the cleft. For example, this might be a null requirement, with no contrariness needed at all, it might be the requirement that a salient individual thinks the contrary proposition is possible, or it might be the requirement that a salient individual is certain of the contrary proposition. Now suppose that our experimental subjects are uncertain as to the exact meaning of a cleft, each entertaining a mixture of base grammars as possible models of the meaning of a cleft, and attributing different probabilities to each base grammar. Imagine that a person—for whom each base grammar *G*_*i*_ is assigned a non-trivial probabilities *p*_*i*_—is faced with an example which is grammatical according to grammars *G*_1_, …, *G*_*r*_, and ungrammatical according to grammars *G*_*r*+1_…*G*_*n*_. Let us suppose that their judgment of the grammaticality will be proportional to $\Sigma_{1}^{r}p_{i}$. That is, we suppose that felicity of an example is proportional to the likelihood of the grammar being one which accepts that example. In that case, the more contrary the context for an example, the more positive will be the predicted felicity judgment, since a more contrary case is bound to satisfy strictly more grammars. Further, the model would allow variation across experimental subjects to be modeled in terms of them having different base grammar probability distributions. Such a model could account for our gradient data entirely in terms of conventionally stipulated, categorical contrariness requirements of clefts. Thus, while we make no claim to have resolved whether contrariness is pragmatic (in which case an explanation of the phenomenon would still be needed), or based on a conventional requirement for contrariness, what we can say is that accounts of the meaning of clefts which are restricted to only the three standard components of cleft meaning are insufficient, since these do not account for our data.

Our research also relates to discussions on the definition of contrast concerned with how to characterize the nature of the alternatives in the interpretation of contrastive focus (as opposed to plain focus, or “informational” focus following É. Kiss, 1998). As discussed in the introduction, the past literature has often identified three relevant ingredients to contrast, namely the size of the alternative set, the identifiability of its elements, and the exclusion requirement of the alternatives. Our study speaks to the role of these aspects in that our experimental design included a **non-contrastive** context, in which these aspects were absent (i.e., an alternative to the focused element was not explicitly mentioned, and therefore what was said about the contrastively focused element potentially held of its alternatives), and **contrastive** contexts, in which the size of the alternative set was restricted to one alternative, explicitly mentioned (thus identifiable), and for which the predicate did not hold. Although we found that clefts' naturalness ratings were significantly better in the latter contexts for both languages, French clefts were rated fairly high in the non-contrastive context. This suggests that the presence of a clearly identifiable alternative (set) is not required in this language—the pivot position does not seem influenced by the alternative type, while it is in English. Thus, the grammatical sensitivity to this particular aspect of contrast differs between French and English.

Our main finding, though, suggests that characterizing contrast solely in terms of contrast set size, element identifiability and the exclusion requirement is insufficient. We have shown that the notion of *contrariness* is also important and indeed better explains clefts' use-conditions, both in French and English. This is where we would like to relate our finding to an idea present in Repp (2016): To gain a precise understanding of the notion of contrast, one should not only consider the way in which alternatives are construed, but also the type of context in which two sentences or discourse segments appear. Put slightly differently, Repp claims that while the alternativeness of constituents has to do with the explicitness (or lack there of) of the alternative (set), another important element of contrast has to do with the type of *discourse relation* in which sentences are involved. While the basic ingredients of contrast are that there must be similarities and dissimilarities between two sentences, Repp also discusses the fact that additional aspects can come into play—e.g., a violation of expectation—that lead to having a different discourse relation between two segments *d1* and *d2*. Repp hypothesizes that three relations are most relevant to the notion of contrast, which she calls non-contrastive, oppose and correction relations. Crucially, she argues that these three discourse relations correspond to increasingly stronger degrees of contrast, which stems from the idea that contrast should indeed be considered a gradable phenomenon (an idea already present in some prior work such as Molnár, 2006). For instance, Repp argues that two segments in a correction relation express contrast more strongly than two segments that stand in an oppose relation. The core difference between the three relations lies in the type of contribution that *d1* and *d2* make to the discourse: while *d1* and *d2* cannot be simultaneously true in an correction relation but can in a non-contrastive one, while they make opposing contribution in an oppose relation.

How do these discourse relations relate to the present work? In our experiment, given Repp's definitions for each relation, our contrastive contexts all involve a correction relation between the discourse segment of Speaker A and B—i.e., a piece of information in A is rejected by B, thus the propositions associated with the two segments cannot be simultaneously true. Therefore, although it would be tempting to try and explain our data in terms of differing discourse relations, our stimuli all stand in one and the same relation of correction. It would also be reasonable to cast an explanation of our data in terms of clefts being inherently corrective rather than inherently contrary. Put slightly differently, our notion of *contrariness* could be seen as an implementation of the notion of *corrective focus*: see e.g., Gussenhoven (2008). However, even though our analysis is inspired by correctivity accounts as well as by Zimmermann's, three differences are worth noting. First and most importantly, we take *contrariness* to be a matter of degree, which is not how corrections are normally analyzed. Indeed, existing accounts of correctivity do not incorporate any notion of degree, whereby one correction is in some sense stronger than another. In Repp's account, for example, the correction relation either holds between discourse segments or fails to hold, with no in between. The degree of contrast is encoded across relations, not within one. In our experiment, we varied the degree of contrast within the relation of correction. Therefore we can say that extant models of correctivity could not account for our data, and such models would have to be augmented in some way that would allow corrections of weakly held beliefs to be differentiated from corrections of strongly held beliefs.

Second, it seems plausible to have instances of contrariness where the claim runs counter to expectation but there has been no explicit counter claim to correct. Consider the example in (15). To deal with this example in a correction-based theory would require some modification to allow for the possibility of correcting things that have not actually been said, for instance by accommodation. Although, we do not dispute that such accommodation will sometimes be needed, we believe this is stretching the notion of correction unreasonably. Moreover, to make it work in Repp's account, which is a discourse-relation based account, one would need accommodation of a contrary utterance. Because our contrariness account is based not on differences between what has been said, but on the difference between beliefs, to account for cases like (15), we require a different type of modification, namely accommodation of contrary belief. We recognize however, that this is a quite subtle difference between Repp's corrective account and our contrariness account; a difference as to whether they focus on what is believed vs. what is said, and that it might be hard to find examples that truly distinguish between the two.

**Table:** 

(15)	A: Who won the NBA dunk contest this year?
	B: No way you'll believe this, but it just so happens that it was an unknown contender from Iowa called Louis D. Johnson who managed to get the most points, and on the final dunk!
	A: No fucking way!
	B: Yes fucking way - you should have seen her alley-oop windmill off the back of a donkey? Johnson is incredible!!!

Third, Prince's informative clefts are a problem for corrective analyses. Consider for instance example (16):

**Table:** 

(16)	It was at the University of Iowa that Camille D. Johnson first managed to apply her deep knowledge of clefts in natural language to the world of particle physics, and, for the first time in human history, to split the atom entirely by the use of carefully targeted questions.

While there is no prior material being corrected here, it is not implausible that, in such cases, the claim is being presented for rhetorical effect as running counter to an expectation. Here again though, it is implausible that we could accommodate that someone had said something contrary to this, but it is quite plausible that we could accommodate a contrary belief or expectation^8^.

Finally, our findings can be considered in the broader light of prior work on the function of prosody and other ways of marking information status. Much prior work on focus has emphasized properties that relate to the presence of some prior structure in discourse, for example the presence of a question, of an element of the same type as the target, or of a clause which exhibits structural parallelism. A different line of work was initiated by Pierrehumbert and Hirschberg (1990), who analyze various types of intonational contour in terms of speaker and hearer expectations. Our experiments and analysis imply that clefts have an intrinsically doxastic function. While the specific results we have obtained are not predicted by any prior model, they do suggest that the Pierrehumbert and Hirschberg approach is on right track for analyzing the marking of information status more generally.

Indeed, they are also in line with work suggesting that marking of speaker expectation is a central function of language, markers of such expectations sometimes being brought together in a (controversial) category of *miratives* (DeLancey, 1997). It is notable that several focus sensitive constructions have been taken to be mirative, including scalar additives like English *even* / French *même* and exclusives like English *only* / French *seulement* (see e.g., Beaver and Clark, 2009). Recently, Cruschina (2012) discusses the relationship between contrast and *focus fronting* (i.e., movement of the focus constituent to the left-periphery of the sentence), arguing that different subtypes of focus are relevant for the realization and interpretation of this syntactic movement. For instance, the author shows that while most Romance languages employ focus fronting as a strategy to signal the most explicit case of contrast, namely *correction*, they also resort to this strategy to encode mirative focus, that is new information that is particularly surprising or unexpected to the hearer. In the same vein, Trotzke (2017) provides empirical evidence for German. Results from an acceptability judgment task suggest that focus fronting in this language is also more commonly associated with a mirative interpretation rather than either a corrective or a contrastive interpretation. Finally, Bianchi et al. (2016) find that the intonational patterns associated with fronted constituents in the mirative condition differs from those found in the correction condition, thus positing that mirative focus is indeed grammatically distinct from corrective focus. The authors go on discussing the nature (or status) of this mirative interpretative effect, analyzing it as a conventional implicature (in the sense of Potts, 2005). Going back to the construction of interest in this paper, the fact that clefts, which help mark focus, turn out to have a function related to speaker expectation is of a piece with the fact that some focus sensitive constructions have previously been identified as mirative. Although we are not currently in a position to make any strong claims about the nature of the contrariness requirement we posit for clefts, we acknowledge that the analysis proposed by Bianchi et al. (2016) for the mirative effects associated with focus fronting might be extendable to clefts. Given the amount of work on the nature of exhaustivity in clefts, which is analyzed as an implicature by some (see e.g., Horn, 1981; Destruel, 2013), one line of research worth pursuing would be to directly compare the strength of the contrariness effect with the effects of classic inferences failing such as exhaustivity.

While our data answers the main questions we set out with, it is also suggestive of new questions. First, we might ask whether the judgment effects we have observed would be mirrored in usage data, e.g., in terms of the frequencies of canonical sentences and cleft sentences in more or less contrary contexts. Indeed, although rating scales tend to provide stable, replicable and transparent pieces of data (Tonhauser and Matthewson, unpublished manuscript), one limitation concerns the possible variation in participants' interpretation of the provided Likert scale, and therefore their resulting use of the scale to provide their judgments. In the present studies, we chose to label each point on the Likert scale rather than only the two end points in order to limit variation as much as possible. Another potential limitation concerns the fact that the language data we are examining involve quite subtle judgments, which might explain the gradience we observe in our results. Given these potential limitations, a corpus investigation would be a welcome addition but such an investigation is not necessarily straightforward and easy to implement, as it would require the operationalization of the notion of contrariness in naturally occurring data. This would certainly be a challenge with a purely automatic methodology for identifying examples in corpora, but perhaps is not beyond what might be achieved using a combination of computational methods for retrieving naturally occurring clefts in context, and human annotation for assessing the degree of contrariness (or, for that matter, correctivity, if this could be assessed as a matter of degree).

Second, for those who accept the premise that gradient data of the sort we see in this experiment is suggestive of a pragmatic rather than a semantic account, what would be the underlying pragmatic explanation? That is, how might one derive from standard assumptions about the meaning of clefts and standard pragmatic principles the fact that clefts are more felicitous as contrariness increases? Finally, given that we have established that in some way clefts are used to mark differences in expectation, how might they be fitted into a more general theory of mirativity, i.e., of how expectation is signaled in human language?

## 4. Conclusions

The goal of the present paper was to test prior hypotheses concerning clefts' standard components of meaning. We hypothesized that the mere presence of an antecedent in discourse which the clefted element would pick up and comment on (i.e., simple contrast) would not suffice to fully explain the felicity pattern of English *it*-clefts. Instead, we set out to test the hypothesis that something more refined is needed, namely a notion of contrast that includes a conflict between interlocutors' expectations. We adapted Zimmermann's notion of contrast, which relates to how strongly the addressee believes the contrary, and experimentally operationalized it. Our data suggests that contrariness does indeed play an important role in helping speakers choose between cleft and canonical forms: the more strongly an interlocutor appears committed to a false proposition, the better it is to repudiate them with a cleft as opposed to using canonical word order, and this effect is visible over and above other factors that distinguish the distribution of clefts in French and English.

## Ethics Statement

This study was carried out in accordance with the recommendations of the IRB at the University of Texas with written informed consent from all subjects. All subjects gave written informed consent in accordance with the Declaration of Helsinki. The protocol was approved by the IRB.

## Author Contributions

ED designed and ran the experiments, collected the data, analyzed the data, and wrote the first draft of the paper. DB helped with ideas in the discussion section and wrote a second draft of that section. EC helped with providing comments on the overall paper, and reworded part of the introduction.

### Conflict of Interest Statement

The authors declare that the research was conducted in the absence of any commercial or financial relationships that could be construed as a potential conflict of interest.

## Footnotes

^1^We further note that our assumptions about the truth-conditional semantics, and indeed about the contrariness implications, are compatible with various explanations given in the literature, which derive the existential and exhaustive implications either by adding presuppositions or through a separate pragmatic process, see for instance (Horn, 1981; Destruel, 2013). Specifically, the conclusions we argue for with regard to contrariness are compatible with a QUD analysis as proposed by e.g., Velleman et al. (2012), but the contrariness results show that the constraints on the QUD would need to be strengthened to account for the requirement that there is a salient contrary view on the correct answer to that expressed by the prejacent.

^2^Throughout the paper, we will indicate ungrammaticality with an asterisk (*) and infelicity with a hash (#).

^3^For É. Kiss (1998), contrast is directly encoded in grammar and realized in a specific sentence position, via clefting in English, and in the left periphery in Hungarian. Put slightly differently, contrast is conventionally encoded in the cleft structure itself. Other scholars, however, argue against the existence of such a strong link, including Horn (1981) and Declerck (1984). On the basis of experimental data in Georgian, Skopeteas and Fanselow (2010a) argue that contrast-related movements are optional, providing evidence that foci realized in the pre-verbal or post-verbal position can receive the same kinds of interpretations (e.g., contrastive or exhaustive). This suggests that contrastivity is not directly encoded in the grammar, at least in Georgian.

^4^Using a 5-point Likert scale, Destruel and Velleman (2014) found that the mean ratings was 1.7 for (5-b), and 2.3 for (6-b).

^5^We discuss how our notion of *contrariness* relates to (accounts of) correctivity in section 3.

^6^Note that we are simplifying in the current paper by only considering cases where the speaker disagrees with the addressee, but a more general definition of contrast would allow for disagreement with third parties.

^7^A reviewer asks why subjects should attribute a stronger commitment to A toward the proposition in context weak than in context no contr.. We believe they should because in context 2, speaker A attributes a particular value, that is identifies a specific referent, that is then contradicted by B.

^8^We contend that to date, no theory that requires an explicit discourse antecedent can account for informative clefts without some additional mechanism of accommodation. However, in augmenting the theory in this way, corrective analyses face a conceptual problem which is absent from the contrariness analysis. The term *correction* is standardly used in linguistics to refer to a speech act used to correct another speech act, or to a rhetorical relation holding between pairs of speech acts. To the extent that our contrariness proposal assumes anaphoricity of the cleft to a discourse antecedent, it faces the same problem. However, the central notion of our contrariness proposal is not disagreement with a speech act, but disagreement with an attitude. To be clearer, there are two distinct requirements in the contrariness theory. If Repp or some other supporter of a correction-based account was to understand the notion of correction more liberally, such that it could include correction of implicit assumptions, then indeed this data would not divide between correction and contrariness accounts. In that case, data like (16) is showing what sort of correction-based account is needed, rather than showing that the notion of correction should be entirely dispensed with. Yet, we believe examples such as (i) might distinguish between the two proposals.

- Emilie (looking disappointed in David): You ate the rest of my cake, didn't you? David (wiping crumbs from mouth and looking guilty): Uhhh, ooops. Yeah, ok, it WAS me who ate it, but I thought you were done, and it looked way too good to waste!

In this type of case, there is clearly no correction since the speaker is agreeing with the suggestion from the speaker's slightly biased question. There is contrariness however, but it is contrariness to norms or expectations about proper behavior, not contrariness with respect to what Emilie said or believed at utterance time. Therefore, our idea is that contrariness is a much more natural notion than correction here. One does not “correct” norms or expectations by showing a counter-instance.

## References

[B1] Atlas J. Levinson S. (1981). It-clefts, informativeness, and logical form: radical pragmatics (revised standard version), in Radical Pragmatics, ed Cole P. (New York, NY: Academic Press), 1–61.

[B2] Barr D. J. Levy R. Scheepers C. Tily H. J. (2013). Random effects structure for confirmatory hypothesis testing: keep it maximal. J. Mem. Lang. 68, 255–278. 10.1016/j.jml.2012.11.001 24403724 PMC3881361

[B3] Bates D. Mächler M. Bolker B. Walker S. (2015). Fitting linear mixed-effects models using lme4. J. Stat. Softw. 67, 1–48. 10.18637/jss.v067.i01

[B4] Beaver D. I. Clark B. Z. (2009). Sense and Sensitivity: How Focus Determines Meaning, Vol. 12 Oxford: John Wiley & Sons.

[B5] Bianchi V. Bocci G. Cruschina S. (2016). Focus fronting, unexpectedness, and evaluative implicatures. Semant. Pragmat. 9, 1–54. 10.3765/sp.9.3

[B6] Buring D. Gutierrez-Bravo D. R. (2001). Focus-related word order variation without the nsr: a prosody-based crosslinguistic analysis, in Syntax at Santa Cruz 3 (Santa Cruz, CA), 41–58.

[B7] Büring D. Kriz M. (2013). It's that, and that's it! exhaustivity and homogeneity presupposition in clefts (and definites). Semant. Pragmat. 6, 1–29. 10.3765/sp.6.6

[B8] Byram-Washburn M. Kaiser E. Zubizarreta M. L. (2013). The English It-Cleft: No Need to Get Exhausted. Boston, MA: Poster, Linguistic Society of America (LSA).

[B9] Carter-Thomas S. (2009). The French *c'est*-cleft: functional and formal motivations, in La linguistique Systémique Fontionnelle et la Langue Française, eds Banks D. Eason S. Ormrod J. (Paris: L'Harmattan), 127–156.

[B10] Cruschina S. (2012). Discourse-Related Features and Functional Projections. Oxford: Oxford University Press.

[B11] Declerck R. (1984). The pragmatics of it-clefts and WH-clefts. Lingua 64, 251–289. 10.1016/0024-3841(84)90065-2

[B12] DeLancey S. (1997). Mirativity: the grammatical marking of unexpected information. Linguist. Typol. 1, 33–52. 10.1515/lity.1997.1.1.33

[B13] Delin J. (1991). Towards a model for generating cleft sentences, in Pragmatics at Issue. Selected Papers of the International Pragmatics Conference (Amsterdam; Philadelphia, PA: John Benjamins), 113–132.

[B14] Destruel E. (2013). An Empirical Study on the Meaning and Use of the French c'est-Cleft. PhD thesis, University of Texas at Austin.

[B15] Destruel E. (2016). Focus marking asymmetries in colloquial and standard french: a stochastic optimality-theoretic account. J. French Lang. Stud. 26, 299–326. 10.1017/S0959269515000265

[B16] Destruel E. DeVeaugh-Geiss J. (2018). On the interpretation and processing of exhaustivity: evidence of variation in English and French clefts. J. Pragm. 138, 1–16. 10.1016/j.pragma.2018.09.009

[B17] Destruel E. Velleman L. (2014). Refining contrast: empirical evidence from the English *it*-cleft, in Empirical Issues in Syntax and Semantics 10, ed Piñón C. (CSSP), 197–214.

[B18] Destruel E. Velleman L. Onea E. Bumford D. Xue J. Beaver D. (2015). A cross-linguistic study of the non-at-issueness of exhaustive inferences, in Experimental Perspectives on Presuppositions, ed Schwarz F. (New York, NY; Dordrecht; London: Springer), 135–156.

[B19] Doetjes J. Rebuschi G. Rialland A. (2004). Cleft sentences, in Handbook of French Semantics, eds Corblin F. de Swart H. (Stanford, CA: CSLI), 529–552.

[B20] Féry C. (2013). Focus as alignment. Nat. Lang. Linguist. Theory 31, 683–734. 10.1007/s11049-013-9195-7

[B21] Gussenhoven C. (2008). Types of focus in english, in Topic and Focus, eds Lee C. Gordon M. Büring D. (Heidelberg; New York, NY; London: Springer), 83–100.

[B22] Halvorsen P.-K. (1978). The syntax and semantics of cleft constructions. (Ph.D. thesis), Austin, TX: University of Texas at Austin.

[B23] Hamlaoui F. (2008). Focus, contrast, and the syntax-phonology interface: the case of French cleft-sentences, in Current Issues in Unity and Diversity of Languages: Collection of the Papers Selected From the 18th International Congress of Linguists (Seoul: Linguistic Society of Korea).

[B24] Harries-Delisle H. (1978). Contrastive emphasis and cleft sentences, in Universals of Human Language, ed Greenberg J. H. (Stanford: Stanford University Press), 419–486.

[B25] Hedberg N. (1990). The discourse function of cleft sentences in English. (Ph.D. thesis), Minneapolis, MI: University of Minnesota.

[B26] Hedberg N. (2013). Multiple focus and cleft sentences, in Cleft Structures, eds Hartmann K. Veenstra T. (Amsterdam: John Benjamins Publishing CompanyJohn Benjamins Publishing Company), 227–250.

[B27] Horn L. (1984). Towards a new taxonomy of pragmatic inference: Q-based and r-based implicatures, in Meaning, Form and Use in Context, ed Schriffin D. (Washington, DC: Georgetown University Press), 11–42.

[B28] Horn L. R. (1981). Exhaustiveness and the semantics of clefts, in Proceedings of NELS 11 (Boston, MA), 125–142.

[B29] Jespersen O. (1927). A Modern English Grammar on Historical Principles: Part VII: Syntax. (Completed and Edited by Niels Haislund). Heidelberg: Carl Winters. (Reprinted 1961 Allen & Unwin, London and Ejnar Munksgaard, Copenhagen).

[B30] Katz J. Selkirk E. (2011). Contrastive focus vs. discourse-new: evidence from prosodic prominence in english. Language 4, 771–816. 10.1353/lan.2011.0076

[B31] Katz S. L. (1997). The syntactic and pragmatic properties of the c'est-cleft construction. (Ph.D. thesis), University of Texas at Austin, Austin, TX.

[B32] Kenesei I. (2006). Focus as identification, in The Architecture of Focus, eds Molnár V. Winkler S. (Berlin: Mouton de Gruyter), 143–159.

[B33] Kiss K. É. (1998). Identificational focus versus information focus. Language 74, 245–273. 10.1353/lan.1998.0211

[B34] Krifka M. (2008). Basic notions of information structure. Acta Linguist. Hung. 55, 243–276. 10.1556/ALing.55.2008.3-4.2

[B35] Lambrecht K. (1987). On the status of SVO sentences in French discourse, in Coherence and Grounding in Discourse, ed Tomlin R. S. (Amsterdam: John Benjamins), 217–262.

[B36] Lambrecht K. (1994). Information Structure and Sentence Form: Topic, Focus and Mental Representations of Discourse Referents. Cambridge: Cambridge University Press.

[B37] Lambrecht K. (2001). A framework for the analysis of cleft constructions. Linguistics 39, 463–516. 10.1515/ling.2001.021

[B38] Lopez L. (2009). A Derivational Syntax of Information Structure. Oxford: Oxford University Press.

[B39] Molnár V. (2002). Contrast from a contrastive perspective. Lang. Comput. 39, 147–162. 10.1163/9789004334250_010

[B40] Molnár V. (2006). On different kinds of contrast, in The Architecture of Focus, eds Molnar V. Winkler S. (Berlin: Walter de Gruyter), 197–234.

[B41] Patten A. (2012). The English It-cleft: A Constructional Account and a Diachronic Investigation. Amsterdam: De Gruyter.

[B42] Percus O. (1997). Prying open the cleft, in Proceedings of the 27th Annual Meeting of the North-East Linguistics Society (NELS). ed Kusumoto K. (Amherst: GLSA).

[B43] Pierrehumbert J. Hirschberg J. (1990). The meaning of intonational contours in the interpretation of discourse, in Intentions in Communication, eds Cohen P. Morgan J. Pollack M. (Cambridge, MA: MIT Press), 271–312.

[B44] Potts C. (2005). The Logic of Conventional Implicatures. Oxford Studies in Theoretical Linguistics. Oxford: Oxford University Press.

[B45] Prince E. F. (1978). A comparison of wh-clefts and it-clefts in discourse. Language 54, 883–906. 10.2307/413238

[B46] R Core Team (2017). R: A Language and Environment for Statistical Computing. Vienna: R Foundation for Statistical Computing.

[B47] Repp S. (2016). Contrast: dissecting an elusive information-structural notion and its role in grammar, in Handbook of Information Structure, eds Féry C. Ishihara S. (Oxford: Oxford University Press), 270–289.

[B48] Rooth M. (1985). Association with Focus. Amherst, MA: Graduate Linguistics Student Association, UMass.

[B49] Rooth M. (1992). A theory of focus interpretation. Nat. Lang. Semant. 1, 75–116. 10.1007/BF02342617

[B50] Sarnicola R. (1988). It-clefts and WH-clefts: two awkward sentence types. J. Linguist. 24, 343–379. 10.1017/S0022226700011828

[B51] Selkirk E. (2008). Contrastive focus, givenness and the unmarked status of ”discourse-new”. Acta Linguist. Hungar. 55, 331–346. 10.1556/ALing.55.2008.3-4.8

[B52] Skopeteas S. Fanselow G. (2010a). Focus in Georgian and the expression of contrast. Lingua 120, 1370–1391. 10.1016/j.lingua.2008.10.012

[B53] Skopeteas S. Fanselow G. (2010b). Focus types and argument asymmetries: a cross-linguistic study in language production, in Contrastive information structure, eds. Breul C. Gobbel E. (Amsterdam; Philadelphia, PA: John Benjamins Publishing Company), 169–197.

[B54] Thoma S. (2009). St'àt'imcets independent pronouns-the invisible cleft, in Proceedings of the 23rd Northwest Linguistics Conference. Working papers of the Linguistics Circle of the University of Victoria (Seattle, WA), 109–123.

[B55] Trotzke A. (2017). Mirative fronting in german: experimental evidence. Rev. Cogn. Linguist. 15, 460–488. 10.1075/rcl.15.2.07tro

[B56] Umbach C. (2004). On the notion of contrast in information structure and discourse structure. J. Semant. 21, 155–175. 10.1093/jos/21.2.155

[B57] Vallduvi E. Vilkuna M. (1998). On rheme and kontrast, in The Limits of Syntax, eds Culicover P. McNally L. (New York, NY: Academic Press), 79–108.

[B58] Velleman D. Beaver D. Onea E. Bumford D. Destruel E. Coppock E. (2012). It-clefts are IT (Inquiry Terminating) constructions, in Proceedings of Semantics and Linguistic Theory (SALT) 22 (Chicago, IL: eLanguage), 441–460.

[B59] Wedgewood D. (2007). Identifying inferences in focus, in On Information Structure, Meaning and Form, eds Schwabe K. Winkler S. (Amsterdam: John Benjamins), 207–227.

[B60] Yasavul M. (2013). Two kinds of focus constructions in k'iche, in Proceedings of the 23rd Semantics And Linguistic Theory Conference, UCSC, CA (Chicago, IL).

[B61] Zerbian S. (2007). Subject/object-asymmetry in Northern Sotho, in Information Structure and the Architecture of Grammar: A Typological Perspective, eds Schwabe K. Winkler S. (Amsterdam: John Benjamins Publishing Company), 323–345.

[B62] Zimmermann M. (2008). Contrastive focus and emphasis, in Acta Linguistica Hungarica is a Quarterly Hungarian Peer-reviewed Academic Journal in the Field of Linguistics, ed Kiefer F. (Akadémiai Kiadó), 347–360.

[B63] Zimmermann M. (2011). The expression of focus in west chadic: variation and uniformity in and across languages. Linguistics 49, 1163–1213. 10.1515/ling.2011.032

[B64] Zimmermann M. Onea E. (2011). Focus marking and focus realization. Lingua 121, 1651–1670. 10.1016/j.lingua.2011.06.002

